# Parametric finite element model of medial patellofemoral ligament reconstruction model development and clinical validation

**DOI:** 10.1186/s40634-019-0200-x

**Published:** 2019-07-05

**Authors:** Vicente Sanchis-Alfonso, Diego Alastruey-López, Gerad Ginovart, Erik Montesinos-Berry, Fabio García-Castro, Cristina Ramírez-Fuentes, Joan Carles Monllau, Angel Alberich-Bayarri, María Angeles Pérez

**Affiliations:** 1 Department of Orthopaedic Surgery, Hospital Arnau de Vilanova, C/Sant Climent, 12, 46015 Valencia, Spain; 2 Multiscale in Mechanical and Biological Engineering (M2BE), Aragón Institute of Engineering Research (I3A), Department of Mechanical Engineering, University of Zaragoza, Zaragoza, Spain; 3 Department of Orthopaedic Surgery, Hospital Terres de l’Ebre, Tortosa, Spain; 4 Agoriaz Orthopaedic Center, Riaz & Clinique CIC, Montreux, Switzerland; 5 QUIBIM SL, Quantitative Imaging Biomarkers in Medicine, Valencia, Spain; 6 Hospital Universitario y Politécnico La Fe and Biomedical Imaging Research Group (GIBI230), IIS La Fe Research Group, Valencia, Spain; 7 Department of Orthopaedic Surgery and Traumatology, Hospital del Mar, Universitat Autònoma de Barcelona, Barcelona, Spain; 8 QUIBIM SL, Quantitative Imaging Biomarkers in Medicine, GIBI230, Biomedical Imaging Research Group, La Fe Health Research Institute, Valencia, Spain

**Keywords:** Patellofemoral joint, Medial patellofemoral ligament, MPFL reconstruction, Finite element methodology, Patellofemoral contact pressure, MPFL-stress

## Abstract

**Background:**

Currently, there is uncertainty regarding the long-term outcome of medial patellofemoral ligament reconstructions (MPFLr). Our objectives were: (1) to develop a parametric model of the patellofemoral joint (PFJ) enabling us to simulate different surgical techniques for MPFLr; (2) to determine the negative effects on the PFJ associated with each technique, which could be related to long-term deterioration of the PFJ.

**Methods:**

A finite element model of the PFJ was created based on CT data from 24 knees with chronic lateral patellar instability. Patella contact pressure and maximum MPFL-graft stress at five angles of knee flexion (0, 30, 60, 90 and 120°) were analysed in three types of MPFLr: anatomic, non-anatomic with physiometric behaviour, and non-anatomic with non-physiometric behaviour.

**Results:**

An increase in patella contact pressure was observed at 0 and 30° of knee flexion after both anatomic and non-anatomic MPFLr with physiometric behaviour. In both reconstructions, the ligament was tense between 0 and 30° of knee flexion, but at 60, 90 and 120°, it had no tension. In the third reconstruction, the behaviour was completely the opposite.

**Conclusion:**

A parametric model of the PFJ enables us to evaluate different types of MPFLr throughout the full range of motion of the knee, regarding the effect on the patellofemoral contact pressure, as well as the kinematic behaviour of the MPFL-graft and the maximum MPFL-graft stress.

**Electronic supplementary material:**

The online version of this article (10.1186/s40634-019-0200-x) contains supplementary material, which is available to authorized users.

## Background

Currently, medial patellofemoral ligament reconstruction (MPFLr) is the “gold standard” in chronic lateral patellar instability surgery. It is typically performed whenever there have been at least two previous episodes of lateral patellar dislocation (Sanchis-Alfonso 2014, 2016). Different surgical techniques with different attachment points, different types of grafts and different configurations for the reconstruction have been described for MPFLr. Each one has good short-term clinical results (Fink et al. 2014; Fulkerson and Edgar 2013; Sanchis-Alfonso 2014; Teitge and Torga-Spak 2004; Weinberger et al. 2017). However, there is uncertainty regarding the long-term outcome of these MPFL reconstructions techniques. To classify a surgical technique for MPFLr as being effective, it is not enough for the instability and pain to disappear. For a surgical technique to be considered effective, new problems like chondropathy or patellofemoral osteoarthritis (PFOA), should never be caused. These problems might be the consequence of the increase in the patellofemoral contact pressure secondary to an inadequate MPFLr (Elias and Cosgarea 2006; Rood et al. 2015; Stephen et al. 2014, 2016), which is clinically relevant because surgery for lateral patellar instability is generally performed in young individuals. Moreover, the development of symptomatic PFOA in young persons does not currently have a good solution. In an ideal MPFLr, the graft should be tense at 0–30° of knee flexion. Beyond 30°, the graft should be loose (Sanchis-Alfonso et al. 2017). All the other parameters should be considered inadequate (Sanchis-Alfonso et al. 2017). Given that in the daily clinical practice many MPFL reconstructions with a clearly incorrect femoral fixation point can be seen, we must evaluate not only the correct reconstructions but also the clearly incorrect ones. An effective way to evaluate patellofemoral contact pressure throughout the range of motion of the knee after MPFLr is by using the Finite element methodology (FEM) (DeVries et al. 2015; Elias et al. 2005; Elias and Cosgarea 2006; Shah et al. 2015). Moreover, this technology also enables us to evaluate the kinematic behaviour of the MPFL-graft and maximum MPFL-graft stress, that is, the tension that the graft can withstand before breaking, in all knee flexion-extension positions.

The objective was to create a parametric model of the patellofemoral joint (PFJ) where the joint geometry is simplified and can be meshed by means of automatic mesh generation programs with suitable finite element aspect ratios for all meshes. Additionally, the aim was that the parametric model would enable a surgeon to simulate different types of surgical techniques for MPFLr. It is hypothesized that this model would allow to evaluate patellofemoral contact pressure and the maximum MPFL-graft stress in each specific reconstruction at different knee flexion-extension angles. The objective was to determine the negative theoretical effects (patellofemoral contact pressure and the maximum MPFL-graft stress) on the PFJ in each type of MPFLr. This negative effect could be related to long-term deterioration of the PFJ.

## Methods

### Parametric finite element model of the patellofemoral joint

**Fig. 1:**
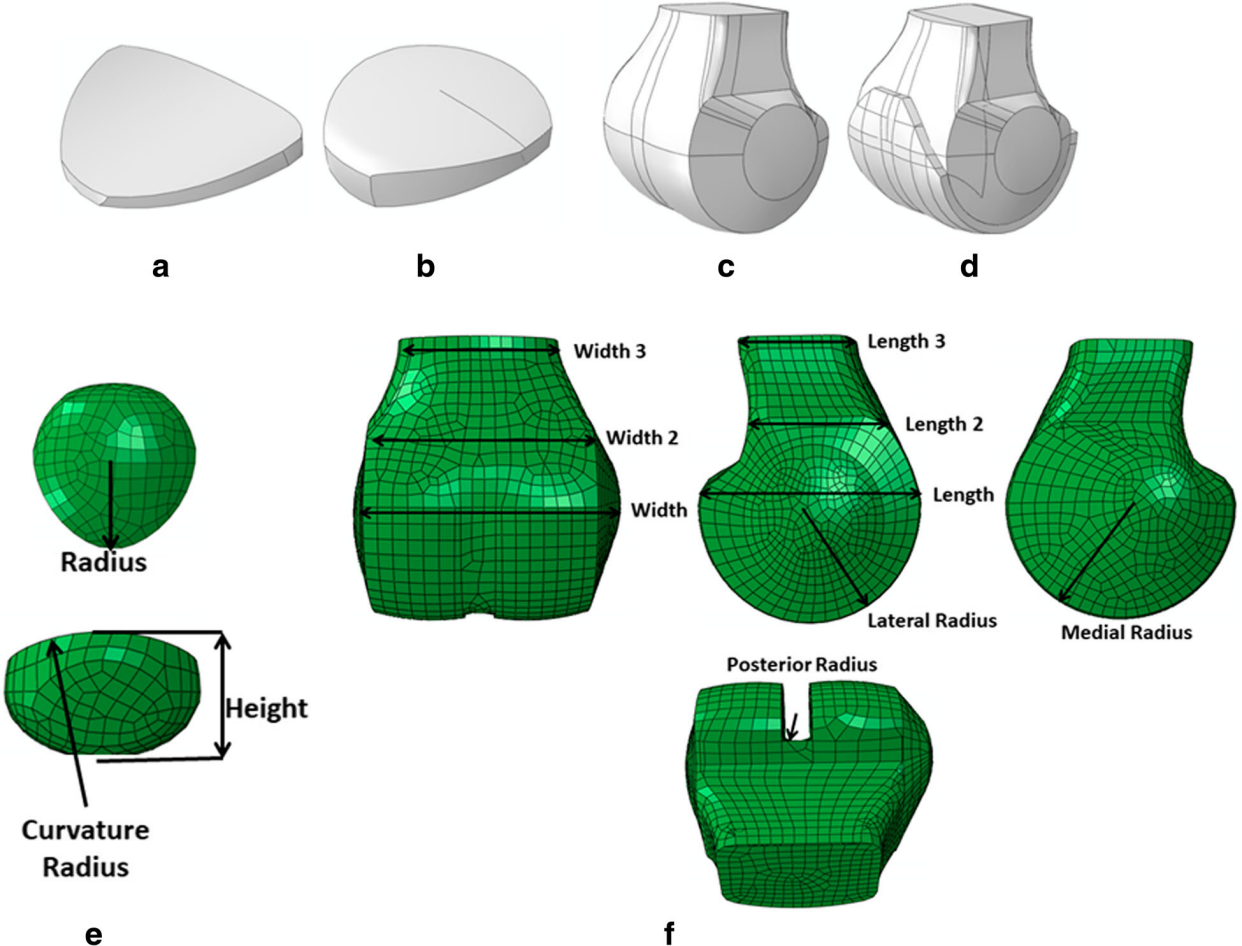
Parametric geometry of the four main parts of the PFJ model: **a** Patellar cartilage; **b** patellar bone; **c** femoral bone; **d** femoral cartilage; **e** geometric parameters of the patellar bone; **f** geometric parameters of the femoral bone

From a previous study (Sanchis-Alfonso et al. 2017), high spatial resolution Computerized Tomography (CT) data were available from 24 knees of patients with chronic lateral patellar instability. Images were acquired with a 64-detector Multi-Detector CT system (Philips Medical Systems, Best, the Netherlands) at the highest spatial resolution, without slice interpolation (0.255 × 0.255 × 0.672 mm^3^). An iterative thresholding scheme was used to extract bones from the imaging data, and triangulated surfaces were defined to describe the outer surfaces (MIMICS, Materialise NV, Leuven, Belgium). The main characteristics and dimensions considered for the parametric model were measured as a reference (femur and patella bone dimensions) from the 24 knees (Sanchis-Alfonso et al. 2017). Knee geometry was simplified to construct a 3D parametric model that achieved nearly anatomical geometry with variable parameters (i.e., trochlear dysplasia, patellar width, patellar diameters, geometry of the patella). The parameters were measured from CT scans both on the axial plane and by using a multi-planar reformatting (MPR) technique. Patients were pathological. Therefore, the parametric geometry also considered their particular geometry. The main parts of the PFJ parametric model were the bones of the femur (femoral condyle) and patella as rigid parts as well as the femoral and patellar cartilages as hexahedral deformable components (Fig. 1a-d). As previously stated, each part was simplified to obtain nearly anatomical geometry with variable parameters (Elias et al. 2016). The patellar bone was modelled starting from a concave-revolution-solid shape, with the parametric radius, height and radius curvature (Fig. 1e). Several revolution cuts were performed on the solid part, and its final geometry was obtained (Fig. 1b). The patellar cartilage was created following the same procedure while maintaining the patellar dimensions (Fig. 1a). The femoral bone was the most complex part of the model. It was defined as a discrete rigid part that had four main elements: a revolution shape that defined the bottom geometry, with a parametric width and radius (lateral and medial); a solid loft for the irregular section, with different width and length parametric sections (width, width 2, width 3, length, length 2, length 3); a revolution shape in the posterior geometry, where the radius can be modified; and two revolution shapes (Fig. 1f) that represent the femoral epicondyles (Fig. 1c). Width and length parameters corresponded to the maximum distance between both femoral epicondyles. Width 2 and length 2 were taken at the point where the medial epicondyle joins the main femoral bone. Width 3 and length 3 were measured at the same point as the highest position of the patella (0° knee flexion angle). The posterior radius defined the contact region between the patellar and femoral cartilages. The femoral cartilage was defined as deformable, and its generation was based on femur geometry and consisted of a revolution shape for the bottom geometry and a combination of elements that defined the upper region (Fig. 1d). The PFJ parametric model was developed using the Abaqus/CAE v.6.14 software (Dassault Systèmes, France). Measuring previous geometrical characteristics on the 24 knees, a mean parametric model was generated (Table 1).

**Table 1:** Mean values (mm) and standard deviation (±) (mm) of the geometrical parameters defining the parametric PFJ measured on the 24 knees (Sanchis-Alfonso et al. 2017)

Femoral geometrical characteristics								
Width	Width 2	Width 3	Length	Length 2	Length 3	Medial radius	Lateral radius	Posterior radius
72.28 ± 8.92	54.00 ± 5.83	39.01 ± 3.85	47.71 ± 5.21	33.99 ± 3.40	28.44 ± 3.00	28.91 ± 4.75	26.09 ± 4.21	13.84 ± 2.71
Patella geometrical characteristics								
Radius			Curvature radius			Height		
20.26 ± 6.24			45.49 ± 3.31			19.09 ± 2.46		

**Fig. 2:**
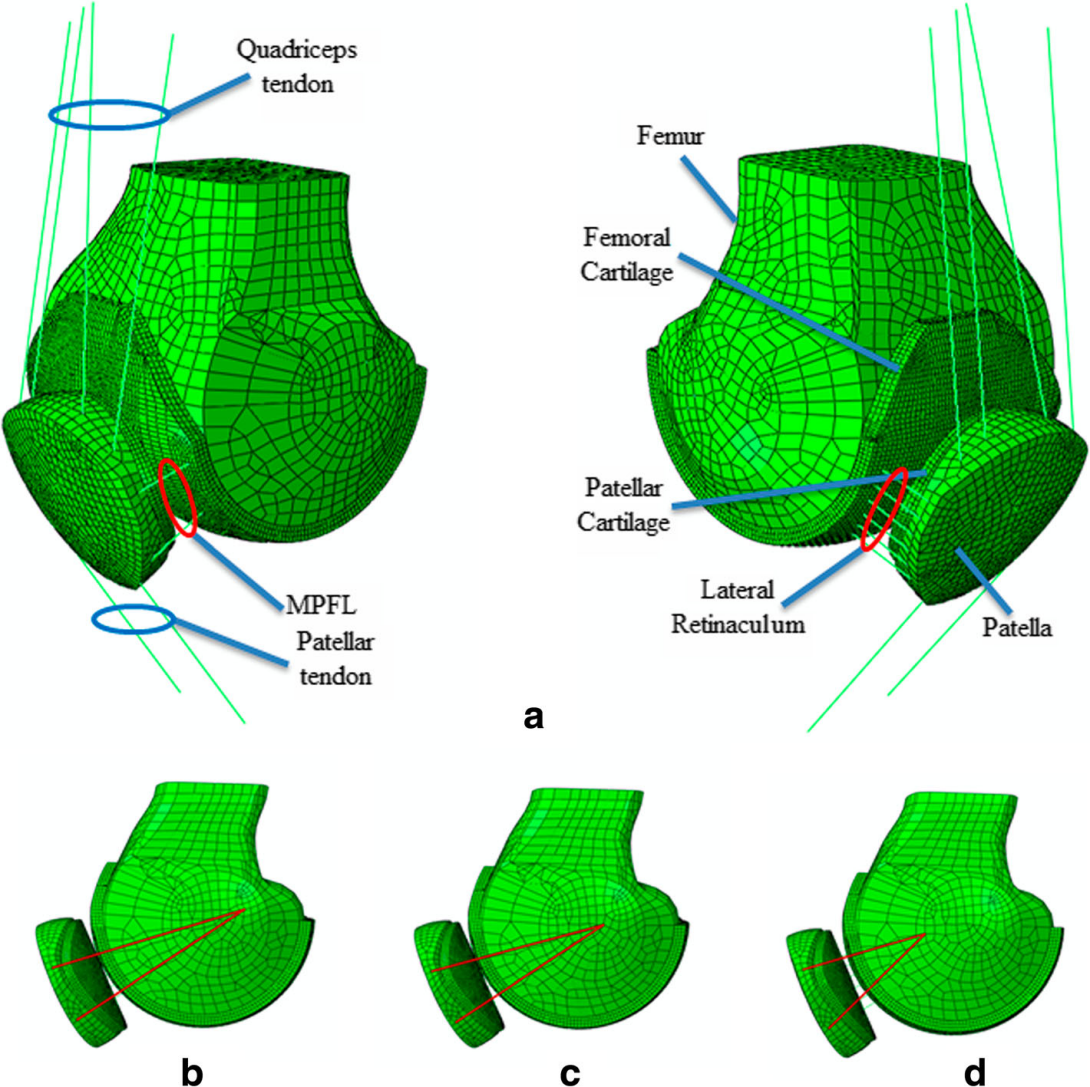
**a** Final model reconstruction including the joint ligaments and tendons. **b** Reconstruction with a patellar bone fixation point and an anatomic femoral fixation point. **c** Reconstruction with a patellar bone fixation point and a non-anatomic femoral fixation point that has physiometric behaviour. **d** Reconstruction with a patellar bone fixation point and a femoral fixation point that is too far anterior and without physiometric behaviour

As cartilages cannot be reconstructed correctly from a CT, a fixed thickness of 3 mm was assumed (Cohen et al. 2003). Tendons and ligaments were also included since they help to stabilize the patella and better distribute patellofemoral pressures (Fig. 2a). The quadriceps tendon (QT), which consists of the vastus medialis (VM), vastus lateralis (VL), vastus intermedius (VI), and the rectus femoris (RF) tendons and the patellar tendon (PT) were modelled as a group of four and two truss elements, respectively (Fig. 2a) whilst the MPFL and the lateral retinaculum (LR) were defined as beam elements (B33) (Fig. 2a). The QT was oriented from the insertion site on the patella to the muscle origin or the most distal wrapping point on the femur. The PT was oriented from the distal patella to the tibia (Elias et al. 2005; Elias and Cosgarea 2006; Elias et al. 2006). The tendon and ligament properties were taken from previous studies (Ciccone II et al. 2006; Drez Jr et al. 2001; Elias and Cosgarea 2006) and are summarized in Table 2. A radius of 1 mm was assumed for the beam elements. A mesh convergence analysis was performed for the deformable parts, which determined that an element size should be 1 mm, so that the cartilages would have at least three elements along their thickness. Finally, the patellar cartilage was compounded by 5756 nodes and 4125 elements, while the femoral cartilage was defined by 24,918 nodes and 18,201 elements. The cartilages were modelled with an elastic modulus of 10 MPa and Poisson’s ratio of 0.45 (Blankevoort and Huiskes 1991; Fernandez et al. 2012; Shah et al. 2015).

**Table 2:** Material properties considered for ligaments and tendons in the FEM simulation

Material Properties		
	Stiffness (N/mm)	Poisson Ratio
Quadriceps Tendon (QT)	1350	0.3
Patellar Tendon (PT)	2000	0.3
Lateral Retinaculum (LR)	2	0.3
Native Medial Patellofemoral Ligament (MPFL)	12	0.3
MPFL Reconstruction (Semitendinosus Graft)	100	0.3
MPFL Reconstruction (Gracilis Graft)	80	0.3
MPFL Reconstruction (Quadriceps Tendon Graft)	33.6	0.3

Bone-cartilage interactions, i.e., femoral bone with femoral cartilage and patellar bone with patellar cartilage, were defined as a tie constraint. The contact between both cartilage surfaces (femoral cartilage with patellar cartilage) was defined as a surface-to-surface standard contact with a contact adjustment of 0.1, a hard contact for the normal behaviour and a penalty friction formulation with a friction coefficient of 0.02 for the tangential behavior (Besier et al. 2008). A sensitivity analysis was performed changing the friction coefficient (see Additional file 1).

### MPFL reconstruction techniques

Three types of MPFL double-bundle semitendinosus reconstructions with patellar and femoral bony attachment were simulated based on a previous study (Sanchis-Alfonso et al. 2017): anatomic reconstruction, meaning a reconstruction with a femoral anatomic fixation point (Fig. 2b); non-anatomic but physiometric reconstruction, meaning the femoral fixation point is not anatomic, but behaves kinematically like a native MPFL (Fig. 2c); and non-anatomic and non-physiometric reconstruction (Fig. 2d). For this last type of reconstruction, the femoral fixation point is too anterior, which means the ligament is too short and that it behaves kinematically, the opposite of a native ligament (Sanchis-Alfonso et al. 2017). The length of a normal MPFL increases during flexion from 0 to 30° and decreases from 30 to 120° (Sanchis-Alfonso et al. 2017). This pattern is considered as the in vivo MPFL standard dynamic length change. In a normal (anatomic) MPFL reconstruction, the graft is isometric in all the cases between 0 and 30° of knee flexion (Sanchis-Alfonso et al. 2017). In 83% of cases, it is isometric from 0 to 60° of knee flexion (Sanchis-Alfonso et al. 2017). Beyond 60° of knee flexion, the MPFL becomes progressively lax and isometry is lost (Sanchis-Alfonso et al. 2017). Regarding isometry, a ligament is considered isometric when there is less than 5 mm of length change throughout the range-of-motion (Smirk and Morris 2003).

### Simulation of the different surgical techniques

The three surgical techniques were analysed for 5 knee flexion positions: 0, 30, 60, 90 and 120°, as in a previous dynamic CT scan study (Sanchis-Alfonso et al. 2017). Initially, for all of the surgical techniques, the patellar group (bone and cartilage) was not in contact with the femoral group (bone and cartilage) to avoid non-desirable initial contact problems. The patella was initially aligned with the trochlear groove using the CT images. A perpendicular displacement (approximately 0.5 mm) to the femoral cartilage surface was imposed upon the patella. Once the contact between both cartilages was generated, initial contact pressures were stored. Then, the ligaments and tendons were included and the three surgical MPFLr techniques were analysed. The elements representing the QT and PT were then fixed so that the model was in equilibrium and no forces were applied through them. The initial contact pressures were subtracted from the ones generated with the ligaments and tendons inclusions. Therefore, the results are presented in terms of relative contact pressures, which we subsequently refer to as the contact pressure, to compare the different surgical techniques under the same conditions. The femur position was fixed once every knee flexion position was simulated.

The data considered for the MPFL inclusion were taken from a previous study (Sanchis-Alfonso et al. 2017). Table 3 summarizes the mean distance between the patella and femoral insertion points for the different MPFL reconstructions. Based on that data, the insertion nodes for each technique and the elongation suffered by the ligaments were determined. The reference position, where the ligaments did not experience any strain was considered knee flexion at 40°. The average MPFL lengths were considered, in this part of the study, to compare the performance of the different surgical techniques over the mean parametric FEM of the PFJ. LR lengths were assumed to be the same as the MPFL length to preserve the equilibrium on both sides of the joint.

**Table 3:** Distance between the patellar and femoral insertion points for the MPFL reconstruction techniques analyzed

Flexion Angle (°)	Anatomic MPFL Reconstruction		Non-anatomic MPFL Reconstruction with Physiometric Behavior		Non-Anatomic MPFL Reconstruction but Non-Physiometric	
	Length (mm)	SD (mm)	Length (mm)	SD (mm)	Length (mm)	SD (mm)
0	60.2^b^	± 6.1	51.6^b^	± 4.6	37.5^b^	± 7.8
30	57.9^b^	± 6.8	50.8^b^	± 5.4	36.5^b^	± 9.2
40	57.7	± 6.0	48.8	± 5.0	36.2^c^	± 8.1
60	57.3^a^	± 6.4	44.9^a^	± 5.2	35.7^c^	± 10.1
90	55.6^a^	± 5.7	38.3^a^	± 4.9	35.6^c^	± 7.9
120	50.7^a^	± 4.9	33.7^a^	± 4.8	35.4^c^	± 5.6

MPFL with non-anatomical femoral attachment point with satisfactory results is always physiometric. MPFL with non-anatomical femoral attachment point with non-satisfactory results is always non-physiometric

^a^No tension

^b^Tension type 1

^c^Tension type 2

The average length of the MPFL for each surgical technique was analysed (Table 3), indicating that the distance between the femur and patella insertion points was smaller than the reference distance (40°) in some knee flexion positions. That means that the ligament is not experiencing any type of stress. Thus, analysis of certain positions was not necessary (Table 3, cases indicated by ^a^). In the remaining positions, two different types of simulations were performed. First, in certain positions, the MPFL undergoes an elongation, which is simulated by applying a pretension force, *∆l* ∗ *K*, where *∆l* is the length increment and *K* is the stiffness of the ligament (Table 3, cases indicated by ^b^). Second, several positions showed an MPFL length that was only possible if the cartilage was compressed because the distance between the patella and femoral insertion point was further than in the reference position (40°). As only-tension elements can be compressed, only working under tension, this relative position change was simulated with a temperature reduction equal to $\Delta  l/{l}_{0_{MPFL}}\ast {\alpha}_{MPFL}$, where *∆l* is the length increment, ${l}_{0_{MPFL}}$ is the initial length of the MPFL and *α*_*MPFL*_ is the assumed thermal dilatation coefficient of the MPFL (0.0005 °C^− 1^). This type of simulation allows cartilages to be modelled in a compressed state. Equilibrium on both sides of the joint was preserved assuming the same *∆l* for the LR ligament and with the inclusion of the *α*_*LR*_ coefficient for the LR, calculated as $\Delta  l/\Delta  T\ast {l}_{0_{LR}}$ (Table 3, cases indicated by ^c^), because *∆T* was the same for the entire model. This was an iterative process in which *∆T* was recalculated until the desired length of the MPFL was achieved.

### Clinical validation of the parametric model

Five patient-specific cases were used for clinical validation of our parametric model. The geometry of each patient was generated by modifying the main knee parameters of the parametric model (femur and patella dimensions – see parametric finite element model of the patella femoral joint section-Fig. 1). Patient-specific geometrical data is indicated in Table 4. MPFLr was simulated depending on patient-specific data. The graft insertion points were based on each patient’s geometry with the help of the corresponding CT data. Each patient underwent a different type of MPFLr. Each specific case was simulated bearing the surgeon’s MPFL measurements in mind, as indicated in Table 5. Moreover, all five cases were clinically evaluated by one of the authors (V S-A).

**Table 4:** Patient-specific geometrical data (Fig. 1). Measurements in mm

	Case 1	Case 2	Case 3	Case 4	Case 5
Width	71.6	89.55	66	65.03	70.5
Width 2	49.71	60.13	50.2	46	53.48
Width 3	41.38	41.97	38.78	31.9	40.72
Length	52.74	48.28	51.25	39.09	47.01
Length 2	32.42	40.16	31.3	30.95	33.05
Length 3	28.75	33.47	25.35	28.66	25.4
Medial radius	27.43	36.94	29.56	27.57	22.28
Lateral radius	27.12	33.47	22.09	23.79	23.18
Posterior radius	14.07	11.2	12.43	14.73	12.09
Radius	19.01	20.32	17.12	16.54	32.23
Curvarture radius	44.94	51.24	42.41	43.51	43.17
Height	19.61	23.02	17.94	20.72	16.22

**Table 5:** Patient-specific data for the model validation

Case	Graft Material Configuration	Measured length for each position (mm)					
		0°	30°	40°	60°	90°	120°
1 Non-Anatomic Femoral Attachment point with Non-Satisfactory Result	Semitendinosus SB	36.3^a^	35.9^a^	36.83	38.7^b^	43.7^b^	46.3^b^
2 Non-Anatomic Femoral Attachment Point with Non-Satisfactory Result	Semitendinosus DB (Proximal)	23.1^a^	33.3^a^	36.33^b^	42.4^b^	46.6^b^	48.6^b^
	Semitendinosus DB (Distal)	25.4^a^	39.7^a^	42.77^b^	48.9^b^	54.3^b^	54.8^b^
3 Non-Anatomic Femoral Attachment Point with Non-Satisfactory Result	Quadriceps Tendon SB	56.2^b^	46.8^b^	43.03	35.5^a^	24.2^a^	22.4^a^
4 Anatomic Femoral Attachment Point with Satisfactory Result	Semitendinosus DB (Proximal)	52.2^b^	51.1^b^	50.17	48.3^a^	41.3^a^	35^a^
	Semitendinosus DB (Distal)	49.9^b^	49.7^b^	48.37	45.7^a^	39.7^a^	35.1^a^
5 Anatomic Femoral Attachment Point with Sastisfactory Result	Semitendinosus DB (Proximal)	56.4^b^	57^b^	55.07	51.2^a^	46.9^a^	42.3^a^
	Semitendinosus DB (Distal)	55.1^b^	56^b^	54.17	50.5^a^	45.8^a^	41.9^a^

Cases # 1, 2 and 3 are non-anatomic and non-physiometric

*SB* Single bundle, *DB* Double bundle

^a^No tension

^b^Tension type 1

## Results

**Fig. 3:**
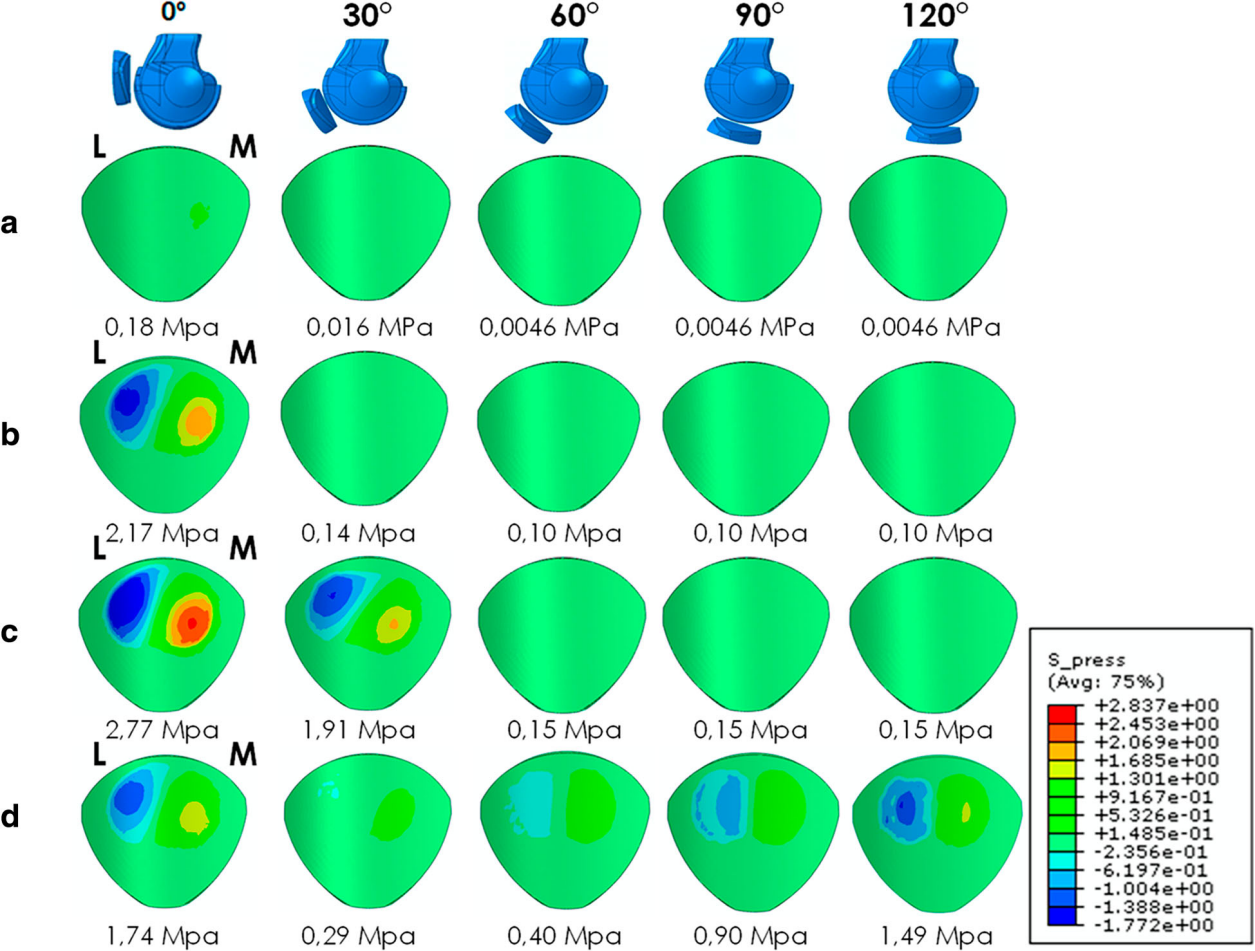
Patellar cartilage contact pressure (MPa): **a** Native knee. **b** Anatomic MPFL reconstruction. **c** Non-anatomic MPFL reconstruction with physiometric behaviour. **d** Non-anatomic MPFL reconstruction with a femoral fixation that is too far anterior and without physiometric behaviour (M-medial; L-Lateral)

In a knee with a virtual intact MPFL, which was used as a reference for the comparison among different reconstruction techniques, the maximum patellar cartilage contact pressures at 60, 90 and 120° were very low compared to the pressures at 0 and 30°. Anatomic reconstruction increased the pressure in all of the knee angles, but the amount of pressure increase was only relevant at 0°. In non-anatomic reconstructions with a physiometric behavior, an increase in all of the positions was found, but the amount of pressure was relevant only at 0 and 30°. In non-anatomic reconstruction without physiometric behavior the pressure increased in all the knee positions and with a relevant amount of pressure. The maximum patellar cartilage contact pressures are displayed in Fig. 3.

In a native knee, both the MPFL and LR are under tension at 0 and 30° of knee flexion. At 60, 90 and 120°, both the MPFL and LR were loose. In both the anatomic and a non-anatomic MPFLr with physiometric behaviour, the ligament was tense between 0 and 30° of knee flexion, but it had no tension at 60, 90 and 120°. In the non-anatomic with non-physiometric behaviour reconstruction, the MPFL was tense at 60, 90 and 120° of knee flexion and was completely loose at 0 and 30° of knee flexion. The MPFL and LR maximum stresses are displayed in Table 6.

**Table 6:** MPFL and LR stress

	Maximum MPFL Stress (MPa)					Maximun LR Stress (MPa)				
	0°	30°	60°	90°	120°	0°	30°	60°	90°	120°
A	8.85	0.78	0	0	0	1.52	0.15	0	0	0
B	74.72	6.55	0	0	0	1.51	0.14	0	0	0
C	97.02	69.60	0	0	0	1.66	1.10	0	0	0
D	63.44	14.74	46.71	77.57	92.70	0.78	0.17	1.24	2.09	2.51

A) Native knee

B) Anatomic MPFL reconstruction with semitendinosus

C) Non-anatomic MPFL reconstruction with a physiometric behavior

D) Non-anatomic MPFL reconstruction with a femoral fixation that is too anterior and without a physiometric behavior

The following cases demonstrate the sensitivity and possible clinical implications of the use of a parametric model of the PFJ using FEM to evaluate MPFL reconstructions.

### Case # 1 (Fig. 4 and Table 7)

**Fig. 4:**
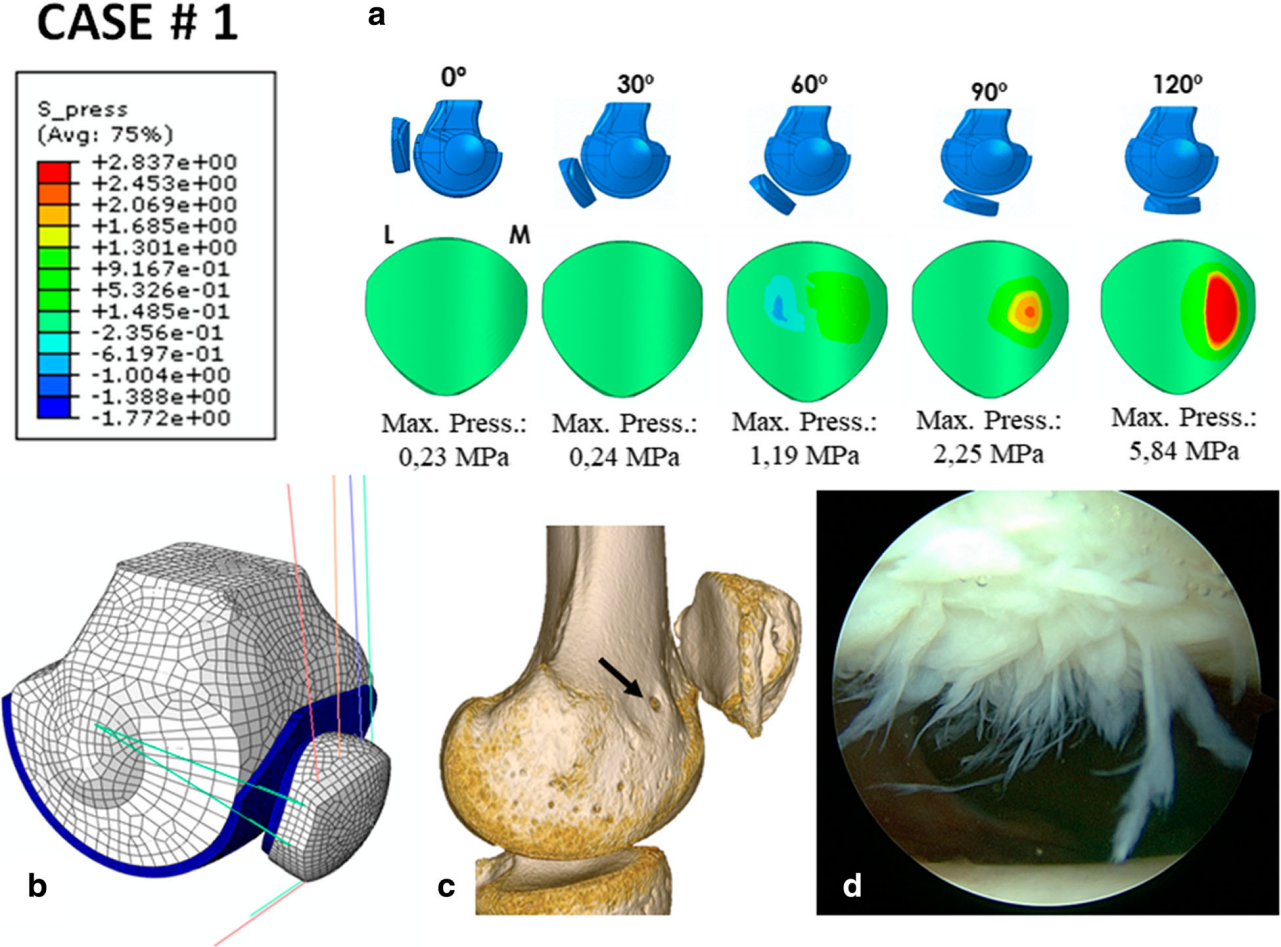
Case # 1 Surgical failure: **a** Contact pressure (MPa) on the patellar cartilage L=lateral, M= Medial. **b** Parametric model of patient # 1. **c** Femoral attachment point is too far anterior (black arrow). **d** Visible patellar chondropathy during arthroscopy

A 17-year-old man was operated on for lateral patellar instability using a single semitendinosus bundle MPFL graft. The patient expressed persistent lateral patellar instability and severe pain. The simulation predicted a contact pressure on the patellar cartilage of 1.19 MPa for 60° of knee flexion, 2.25 MPa for the 90° position and an important contact pressure of 5.84 MPa for 120° of knee flexion (Fig. 4). The maximum MPFL stress at 60° was 59.03 MPa. At 90°, it was 119.2 MPa and 252 MPa at 120°. At 0 and 30°, the MPFL was loose. The maximum lateral retinaculum (LR) stress at 60° was 1.62 MPa, 5.38 MPa at 90° and 7.06 MPa at 120°. At 0 and 30°, the LR was loose. From that data, we predicted that the patient would develop patellar chondropathy, which was in fact seen during the arthroscopy performed during the MPFL revision surgery (Fig. 4d). The tension pattern of the MPFL graft is typically seen in a non-anatomic femoral fixation point that is too far anterior in which the graft exhibits non-physiometric behaviour. This can very clearly be seen in the last preoperative 3D CT scan (Fig. 4c).

**Table 7:** MPFL and LR ligaments stress obtained for each reconstruction and position analyzed

Case	Flexion Angle (°)	MPFL stress (MPa)	LR stress (MPa)
1	60	59.03	1.62
	90	119.20	5.38
	120	252.00	7.06
2	60	19.51	4.56
	90	29.52	7.54
	120	34.7	8.37
3	0	12.28	8.22
	30	3.93	2.68
4	0	60.02	1.15
	30	29.47	0.79
5	0	40.24	0.53
	30	70.30	1.27

### Case # 2 (Fig. 5 and Table 7)

**Fig. 5:**
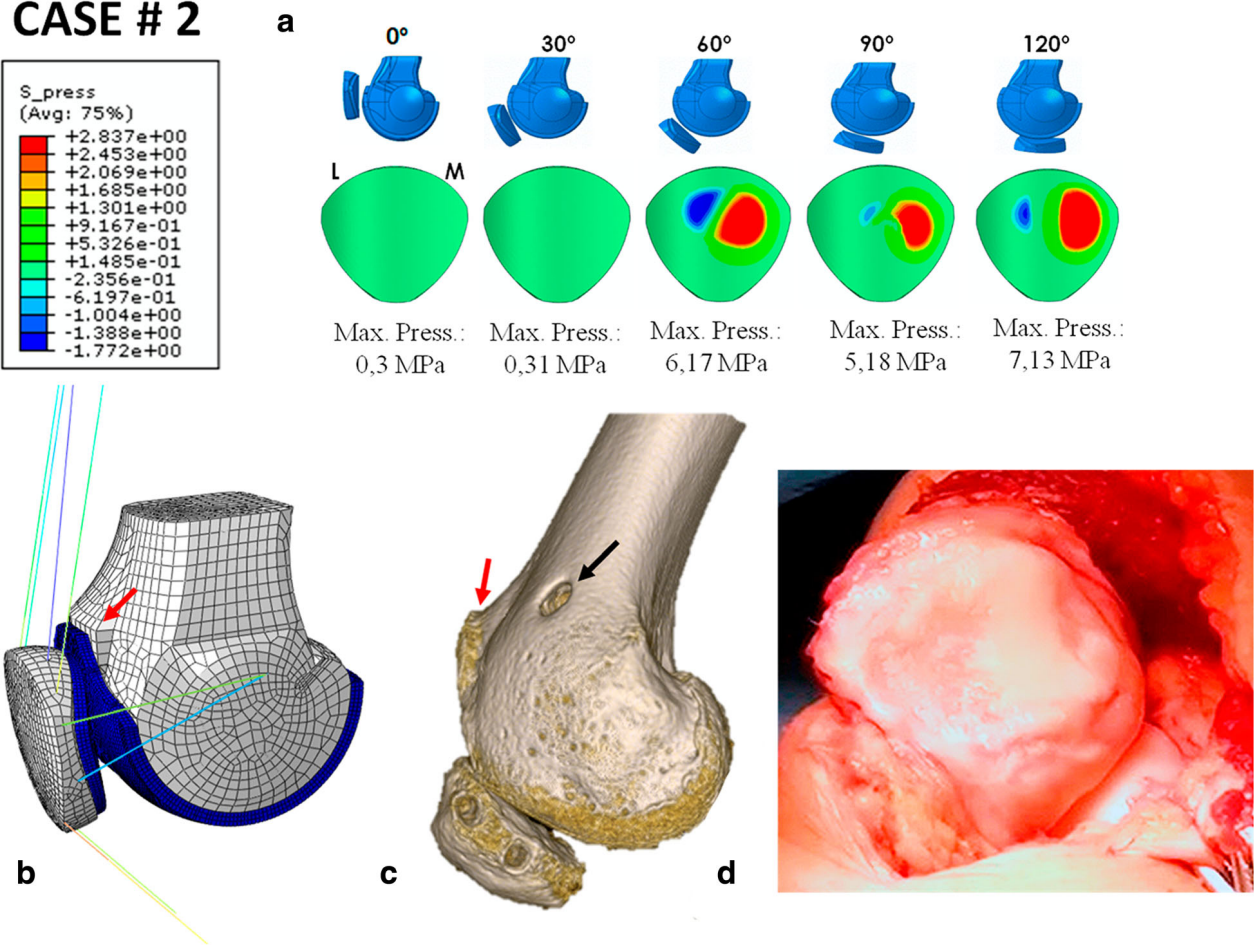
Case # 2 Surgical failure: **a** Contact pressure (MPa) on the patellar cartilage L = Lateral M = Medial. **b** Parametric model of patient # 2. Trochlear dysplasia type D (red arrow). **c** Superior femoral attachment point is too far anterior (black arrow) Trochlear dysplasia (red arrow). **d** Visible patellofemoral osteoarthritis

A 28-year-old woman operated on for lateral patellar instability with a double-bundle MPFL plasty, using the semitendinosus. The patient complained of severe pain and incapacitating lateral patellar instability. The simulation predicted higher contact pressures than in the previous simulation: 6.17 MPa for the 60° knee flexion position, 5.18 MPa for the 90° knee flexion position and 7.13 MPa for the 120° knee flexion position (Fig. 5). The maximum MPFL stress at 60° was 19.51 MPa, 29.52 MPa at 90° and 34.7 MPa at 120°. At 0 and 30°, the MPFL was loose. The maximum LR stress at 60° was 4.56 MPa, 7.54 MPa at 90° and 8.37 MPa at 120°. At 0 and 30°, the LR was loose. The MPFL was tense at 60, 90 and 120° of knee flexion and was completely loose at 0 and 30° of knee flexion. Clinically, this tension pattern will lead to PFOA, which was in fact seen during surgery (Fig. 5d). This tension pattern is typical of a non-anatomic femoral fixation point that is far too anterior, as clearly seen in the 3D CT scan in which the graft exhibits non-physiometric behaviour (Fig. 5c).

### Case # 3 (Fig. 6 and Table 7)

**Fig. 6:**
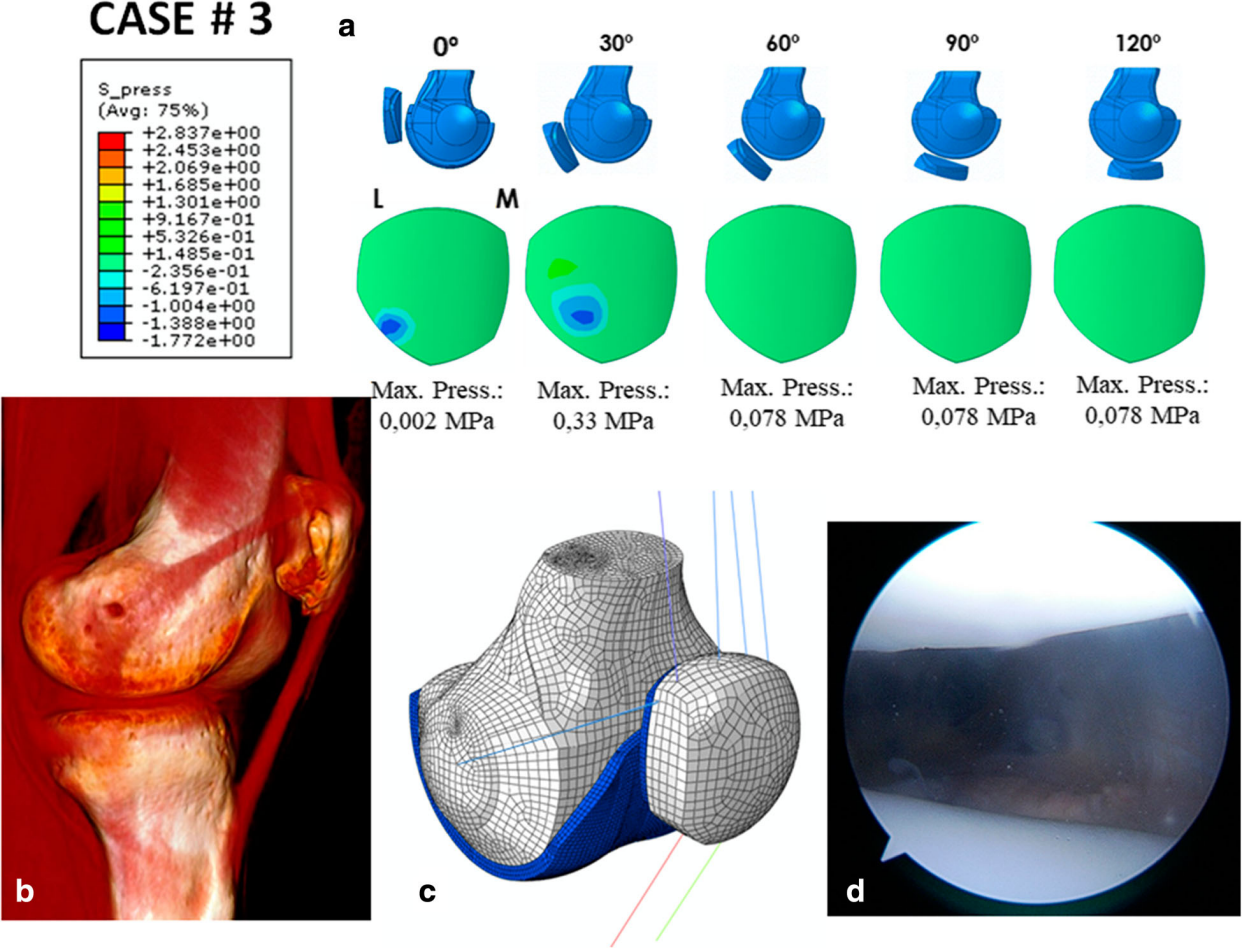
Case # 3 Surgical failure: **a** Contact pressure (MPa) on the patellar cartilage L = Lateral M = Medial. **b** One can see that the graft is preserved; however, the orientation is too oblique and extremely vertical. **c** Parametric model of patient # 3. **d** Arthroscopy at the time of the revision surgery shows normal patellofemoral cartilage

A 38-year-old woman was operated on for lateral patellar instability with an MPFL single-bundle reconstruction using the quadriceps tendon. The patient complained of severe pain and incapacitating lateral patellar instability. The simulation performed with our FEM showed patellofemoral contact pressures far below those found in a native knee (Fig. 6a). The maximum MPFL and LR stresses predicted for the 0° knee flexion position were 12.28 MPa and 8.22 MPa, respectively. They were 3.93 MPa and 2.68 MPa for 30°, respectively. The prediction fulfils the requirements for an effective MPFLr: a tense graft at 0 and 30° of knee flexion, with greater stress than a native MPFL, and the patellofemoral pressure was below the normal values that could cause symptomatic PFOA. In fact, no chondropathy was seen in this patient during the arthroscopy performed in the revision surgery (Fig. 6d).

### Case # 4 (Fig. 7 and Table 7)

**Fig. 7:**
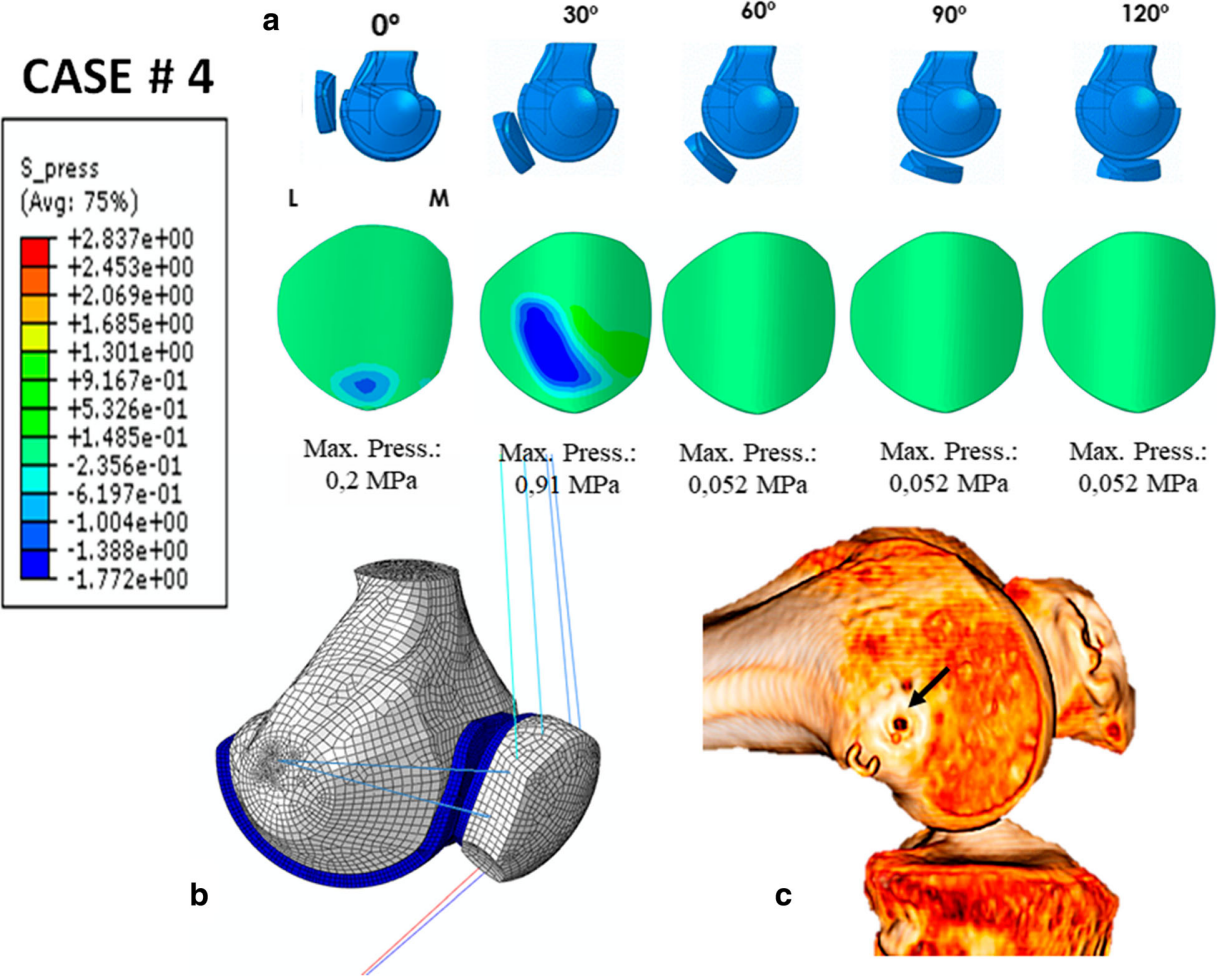
Case # 4 Primary surgery with an excellent result: **a** Contact pressure (MPa) on the patellar cartilage L = Lateral M = Medial. **b** Parametric model of patient # 4. **c** Anatomic femoral attachment point (black arrow)

An 18-year-old woman was operated on for lateral patellar instability with an anatomic MPFL reconstruction using a double-bundle semitendinosus autograft, with an excellent clinical result at 5 years of follow up. The simulation predicted a contact pressure of 0.2 MPa at 0° of knee flexion and 0.91 MPa at 30° of knee flexion. The maximum MPFL and LR stresses predicted for the 30° of knee flexion position were 29.47 MPa and 0.79 MPa, respectively. For 0° of knee flexion, they were 60.02 MPa and 1.15 MPa, respectively. The prediction fulfils the requirements for an ideal MPFLr; a tense graft at 0 and 30° of knee flexion with far greater stress to failure than a native ligament. The patellofemoral pressures were kept below the values that could cause symptomatic PFOA. This tension pattern is typical of an anatomic femoral fixation point as is clearly seen in the 3D CT scan (Fig. 7c).

### Case # 5 (Fig. 8 and Table 7)

**Fig. 8:**
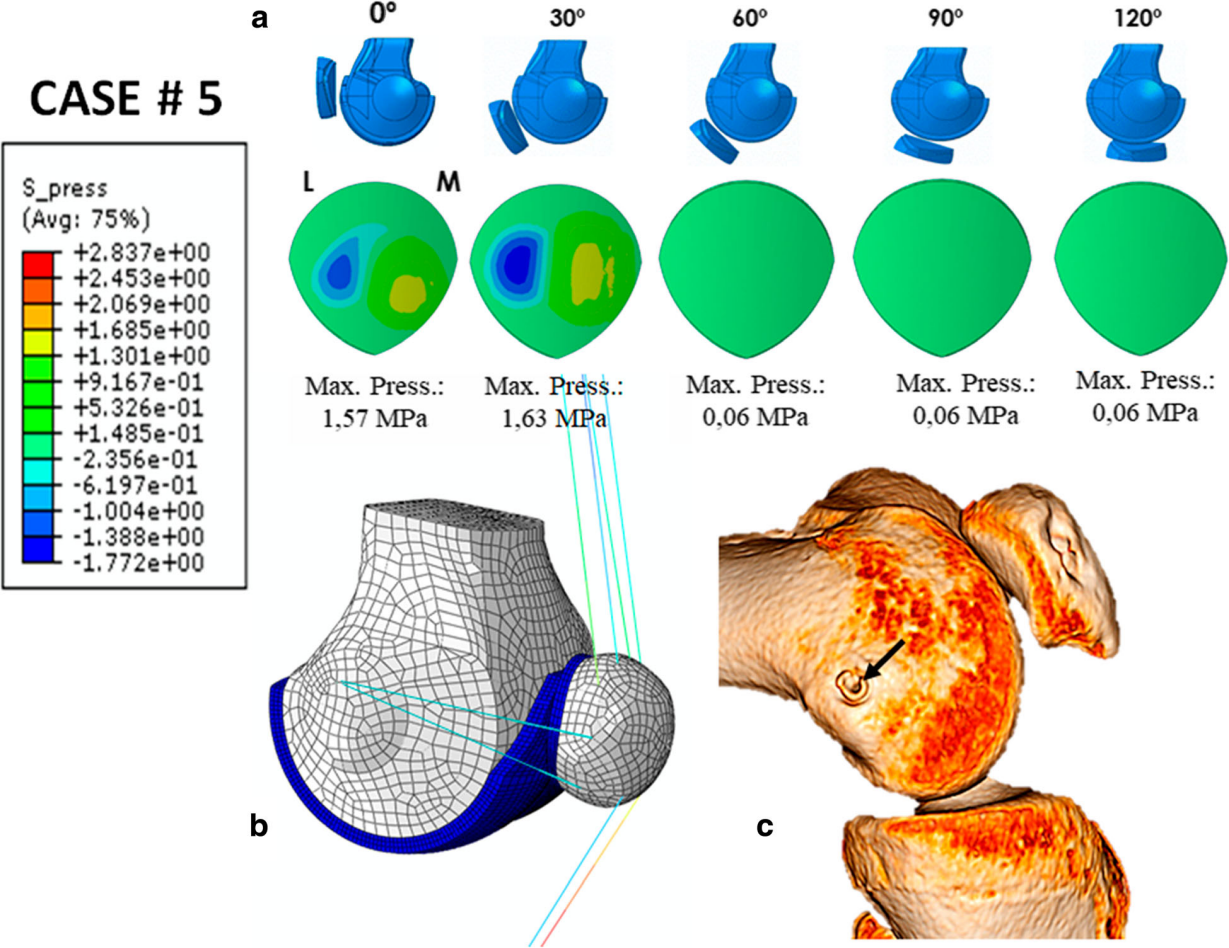
Case # 5 Primary surgery with an excellent result: **a** Contact pressure (MPa) on the patella cartilage L = Lateral M = Medial. **b** Parametric model of patient # 5. **c** Anatomic femoral attachment point (black arrow)

A 15-year-old woman was operated on for lateral patellar instability with an anatomic MPFL reconstruction using a double-bundle semitendinosus autograft, with an excellent clinical result at 5 years of follow up. The simulation predicted a contact pressure of 1.57 MPa for 0° of knee flexion position and 1.63 MPa for 30° of knee flexion position. The maximum MPFL and LR stresses predicted for the 30° knee flexion position were 70.3 MPa and 1.27 MPa, respectively. At 0° of knee flexion, they were 40.24 MPa and 0.53 MPa, respectively. The prediction fulfils the requirements for an ideal MPFLr; a tense graft at 0 and 30° of knee flexion with a far higher stress to failure than a native ligament. The patellofemoral pressure values were below those thought to cause a symptomatic PFOA. This tension pattern is typical of an anatomic femoral fixation point as is clearly seen in the 3D CT scan (Fig. 8c).

#### Model’s accuracy

FEM was very accurate in cases 1, 2, 4 and 5, but not in case 3. Case 3 fulfilled the requirement for a correct plasty relative to the maximum stress and patellofemoral pressure. However, the patient had pain and instability after surgery. The instability could be explained by the single-bundle configuration of the graft, the vertical direction of the graft because of the non-anatomic femoral fixation point (Fig. 6b) and the patella alta. All of them make this graft non-functional. All these factors can contribute to instability and therefore to pain.

## Discussion

This model is the first parametric 3D FEM of the PFJ that analyses the effect of different MPFL reconstructions on the patella contact pressure as well as on the kinematic behaviour of the MPFL-graft and MPFL-graft stress along the total range-of-motion of the knee.

Generation of a patient-specific FEM (i.e., a real FEM) of the PFJ requires CT images to be processed, segmented and then converted into a 3D finite element model. This process is complex, expensive and very time-consuming. However, the parametric model is the opposite. Segmentation is a process that requires manual correction to eliminate undesired tissues, and the computational burden makes the real model unsuitable for clinical integration as a tool for MPFLr planning. A parametric model is a generic model, that is a simplified model valid for any knee that could have direct clinical application.

The difference between the current work and previous ones is that in this paper the contact pressures for all the angles of knee flexion (from 0 to 120°) in both anatomical and non-anatomical (physiometric and non-physiometric) MPFL reconstructions are analysed. Since this is a novel method, we focused on clinical validation. In this way, five clinical cases are presented to demonstrate the accuracy of the model and to show its versatility for predicting challenging clinical cases. An extrapolation of the computational results was performed to provide a qualitative comparison to the clinical outcomes. The contribution of these results is the introduction of FEM in daily clinical practice to optimize surgical procedures by using personalized treatments.

Findings using the FEM are in agreement with those reported in previous computational studies (Elias et al. 2005; Elias and Cosgarea 2006; Shah et al. 2015) and could have meaningful potential implications for clinicians performing MPFLr surgery (Conlan et al. 1993; Desio et al. 1998; Elias and Cosgarea 2006; Hautamaa et al. 1998; Sanchis-Alfonso et al. 2017; Servien et al. 2011). Elias et al. evaluated medial patellofemoral cartilage overload in cases with technical errors during MPFLr estimating contact pressures between 3 and 6 MPa (Elias and Cosgarea 2006; Elias et al. 2016). Shah et al. also obtained very similar values to previous computational studies. Various authors have demonstrated that the changes in the length of a ligament that occur during joint flexion-extension show changes in the tension of that ligament (Good 1995; Moritomo et al. 2009; Sanchis-Alfonso et al. 2017; Seo et al. 2014; Tan et al. 2011). Based on this observation, in a previous study using a dynamic CT scan, it has been concluded that the native MPFL was tense during the first 30° of knee flexion in all cases and progressively loosened after 30° (Sanchis-Alfonso et al. 2017). The explanation behind this conclusion lies in the fact that the attachment points of the MPFL are separated further during the first 30° of knee flexion and become progressively closer from 30° onwards. It is called the physiometric behaviour of the ligament. The current study enabled us to directly confirm these findings. The ligament is tense between 0 and 30° of knee flexion, but at 60, 90 and 120°, it has no tension. This fact has clinical relevance as the MPFL is a structure that is only involved in the lateral stability of the patella during the first 30° of knee flexion. After 30°, the ligament loosens and the patellofemoral contact pressure, which also contributes somewhat to patellofemoral stability and is already low during the first 30° (0.23 MPa), decrease considerably (0.0046 MPa). This finding is in agreement with several anatomic and biomechanical studies that show that the MPFL is the most important restraint to lateral patellar displacement from 0 to 30° of knee flexion (Conlan et al. 1993; Desio et al. 1998; Hautamaa et al. 1998). After 30° of knee flexion, lateral patella stability depends on the femoral trochlea. Additionally, this study confirms previous findings that show that the location of the femoral attachment point is of utmost importance to obtain satisfactory clinical results (Sanchis-Alfonso et al. 2017). The femoral attachment point is related to the patellofemoral contact pressure, tension of the MPFL-graft and physiometry of the reconstruction.

The ideal MPFLr technique must combine a precise balance between an optimal patellofemoral pressure with maximum graft stress. It makes a new tear less likely. The patellofemoral contact pressure of a virgin knee must be reproduced, and a maximum MPFL-graft stress greater than that of the native MPFL must be created with the intention to compensate for the anatomic factors (increased tibial tuberosity – trochlear groove (TT-TG) distance, patella alta and trochlear dysplasia) that predispose to lateral patellar dislocation (Sanchis-Alfonso 2014). In fact, the maximum MPFL-graft stress in both anatomic and non-anatomic but physiometric reconstructions is much greater than that of a native MPFL. However, it is very important not to increase maximum MPFL-graft stress with a subsequent increase in the patellofemoral pressure because the technique will have a suitable result in the short term but will have a deleterious effect and will lead to degenerative changes in the long term. MPFLr evaluation by means of the FEM is more sensitive than evaluations using only clinical or radiological tests. The FEM can demonstrate the validity of a surgical technique in the long term since it enables one to determine whether a specific technique will lead to an increase in the patellofemoral pressure, which is closely related to future development of PFOA. The elevated MPFL graft tension or an incorrect femoral tunnel position will increase the pressure applied to patellofemoral cartilage (Stephen et al. 2014, 2016), and this increase in PFJ contact pressure might result in joint degeneration (Rood et al. 2015; Stephen et al. 2014). Rood et al. in 2015 have shown that static MPFL reconstructions (i.e., reconstruction with both femoral and patellar osseous attachments) result in higher patellofemoral pressures compared with those in the intact situation and thus increase the chance of PFOA in the long term. While Rood et al. showed elevated contact pressures with MPFLr, Stephen et al. in 2014 did not show a pressure increase. In this way Stephen et al., in 2014 and 2016, found that an anatomic MPFLr with a tension of 2 N and fixed at 0, 30 or 60° of knee flexion, regardless of the type of graft used, restores PFJ contact pressures to the intact state. However, graft overtensioning and/or non-anatomic positioning of the femoral attachment increases PFJ contact pressures (Elias et al. 2016; Stephen et al. 2014, 2016). A broad variability in patellofemoral anatomy, graft tension and non-anatomic femoral attachment could explain these different findings.

The current tendency is to perform MPFL reconstructions with an anatomic femoral bone attachment and patellar bone attachment. In our study, we observed an increase in the patellofemoral contact pressures at 0 and 30° of knee flexion after an MPFL reconstruction (2.17 MPa at 0° and 0.14 MPa at 30° when using the semitendinosus as a graft) compared to the pressure found in a normal non-operated knee (0.18 MPa at 0° and 0.016 MPa at 30°). This leads us to consider the possible long-term effects from a slightly greater patellofemoral contact pressures. However, in theory, the patellofemoral contact pressures found in the anatomic reconstructions are not great enough to cause symptomatic PFOA since they are lower than those causing knee osteoarthritis (Segal et al. 2009). The objective would be not to exceed safe levels of patellofemoral pressure to induce patellofemoral chondropathy and ultimately PFOA. It should also be remembered that the increase in patellofemoral contact pressures helps to stabilize the PFJ. Therefore, this factor would be beneficial in the classical anatomic reconstruction. Thus, a discrete increase in contact pressure, as we have observed, is desirable.

Currently, what is being discussed is the precise consequences of the clinical results of the non-anatomical techniques for the MPFLr in which the MPFL-graft behaves like a native MPFL (physiometric behaviour) from the physiological point of view. Servien et al. in 2011 and Sanchis-Alfonso et al. in 2017 found no negative clinical effects after 2 years when using these reconstructions, which could be due to the short follow-up in both cases. In this type of reconstruction, the FEM shows an increase in patellofemoral contact pressure at 0 and 30° of knee flexion in comparison to these pressures in the native knee (2.77 MPa at 0° and 1.91 MPa at 30° vs 0.18 MPa at 0° and 0.016 MPa at 30°). This pressure increase mainly occurs on the medial patellar facet. According to Jones et al. (2016), the average contact stress at 30° is 1.7 ± 0.6 MPa, with a peak of 3.2 ± 0.6 on the surface of the patellar cartilage and of 2.8 ± 0.7 MPa at the deepest point. The differences found between this study and the one by Jones et al. in 2016 can be explained by the fact that Jones uses a laboratory controlled study with cadaver knees using a different method than us. What is not known is whether this pressure increase will result in chondropathy in the long-term and ultimately result in symptomatic PFOA. As far as we know, there is no study of the PFJ that has determined the contact stress threshold that is predictive of symptomatic PFOA. Segal et al. in 2009 observed that a threshold of 3.42 to 3.61 MPa had a 73.3% sensitivity with specificity ranging from 46.7% to 66.7% for the prediction of symptomatic knee osteoarthritis. Obviously, these values cannot be extrapolated to the PFJ, which is the joint with the thickest cartilage in the human body. It is logical to think that the pressures causing symptomatic PFOA would be greater. In non-anatomical MPFL reconstructions, the maximum patellofemoral contact pressures are on the order of 2.77 MPa, values that are considerably below the cut-off point mentioned above. Therefore, it is likely that a non-anatomical but physiometric reconstruction would not have long-term negative effects on the PFJ. Consequently, it would seem more important for the ligament to be “physiometric” rather than perfectly anatomical.

With the FEM, it is possible to predict which MPFLr have an increased risk of severe patellofemoral chondropathy resulting in symptomatic PFOA and requiring active treatment. In the cases in which PFOA occurred, it was because the MPFL-graft was loose, with knee flexion from 0 to 30°, and was tense from 60° onward. In these cases, the patellofemoral contact pressures were over 5 MPa from 60° onward, the femoral attachment point being extremely non-anatomical (too far anterior) and the MPFLr was not physiometric. The predictive value of the parametric model of the PFJ has made its clinical validation possible.

A limitation of this study is that the patellar and femoral cartilages had a constant thickness of approximately 3 mm. The PFJ was reconstructed from CT data in which soft tissues are not clearly distinguished. However, the gap between both bones was approximately 6 mm. Accordingly, the same thickness for both cartilages was assumed. Small differences would have been predicted if other thickness values had been considered. Additionally, the ligament material properties were taken from the literature (Ciccone II et al. 2006; Drez Jr et al. 2001; Elias and Cosgarea 2006). In the future, patient-specific material properties should be considered. The inclusion of magnetic resonance (MR) data from the same patients and the use of image registration techniques might combine MR and CT data. It which would not only make it possible to extract cartilage thickness accurately but also to determine patient-specific multi-variate matrix properties, such as the T1 or T2 relaxation times, which are related to proteoglycan and collagen matrix integrity, respectively (Martí-Bonmatí et al. 2008). Another limitation is that there was no estimation of the amount of error in the patient-specific shape when creating the patient-specific model. There was only qualitative assessment of the global patient-specific shape. Additionally, to preserve equilibrium, the elements representing the QT and PT were fixed and no forces were applied through them. Furthermore, the same LR length changes were assumed as for the MPFL. Another important limitation of this study is the fact that the patellofemoral pressure values that predict the development of a symptomatic PFOA are not known. We have extrapolated the well-known values that would lead to the development of a symptomatic tibiofemoral osteoarthrosis. It has also been hypothesized that the values necessary to develop a symptomatic PFOA should be higher than those for a symptomatic tibiofemoral osteoarthritis because the patellar cartilage is much thicker than that found on the tibia or in the femur (Segal et al. 2009). For that reason, we speculate that a higher pressure would be necessary to cause damage. Using the FEM allows to reliably predict the clinical evolution of an MPFL-graft. Logically, in a condition with multifactorial etiopathogeny such as lateral patellar instability, the model fails in some cases because there are additional factors (e.g., patella alta, increased tibial tubercle-trochlear groove distance and trochlear dysplasia) other than the tension of the MPFL-graft and patellofemoral contact pressures that could be responsible for the failed surgery. This is a major limitation of this study. The abovementioned anatomic additional factors are often associated in patients requiring MPFLr and can change the pressures at the PFJ and lead to different outcomes. Although it has not been addressed in the present work, the conditions in which the graft would not prevent post-operative instability could be incorporated (Farahmand et al. 1998; Hautamaa et al. 1998; Sanchis-Alfonso 2014).

## Conclusion

The main finding of this study is that the use of a parametric 3D finite element model of the PFJ allows the evaluation of different types of surgical techniques for MPFLr with regard to the effect on the patellofemoral contact pressure. That also goes for the kinematic behaviour of the MPFL-graft with flexion-extension of the knee and the maximum MPFL-graft stress based on a previous study which has shown that the graft length variation differs in each type of MPFLr. In this way, from diagnostic images like a CT, for example, it is possible to simulate different surgical treatments and customize the treatment for individual patients.

## Additional file

Additional file 1: **Figure S1.** Patellar cartilage contact pressure (MPa) obtained for different values of friction coefficient (µ) (0.01, 0.02, 0.025, 0.03) in the 30º knee flexion angle for the native knee model. **Figure S2.** Patellar cartilage contact pressure (MPa) obtained for different patellar-femoral cartilages combinations in the 30º knee flexion angle for the intact knee model. PCT=Patellar Cartilage Thickness, FCT=Femoral Cartilage Thickness. (DOCX 387 kb)

## Abbreviations

CT: Computerized Tomography
FEM: Finite element methodology
LR: Lateral retinaculum
MPa: Megapascal
MPFL: Medial patellofemoral ligament
MPFLr: Medial patellofemoral ligament reconstruction
MR: Magnetic resonance
PFJ: Patelofemoral joint
PFOA: Patellofemoral osteoarthritis
PT: Patellar tendon
QT: Quadriceps tendon
RF: Rectus femoris
VI: Vastus intermedius
VL: Vastus lateralis
VM: Vastus medialis

## References

[CR1] Besier TF, Gold GE, Delp SL, Fredericson M, Beaupre GS (2008). The influence of femoral internal and external rotation on cartilage stresses within the patellofemoral joint. J Orthop Res.

[CR2] Blankevoort L, Huiskes R (1991). Ligament-bone interaction in a three dimensional model of the knee. J Biomech Eng.

[CR3] Ciccone WJ, Bratton DR, Weinstein DM, Elias JJ (2006). Viscoelasticity and temperature variations decrease tension and stiffness of hamstring tendon grafts following anterior cruciate ligament reconstruction. J Bone Joint Surg Am.

[CR4] Cohen ZA, Mow VC, Henry JH, Levine WN, Ateshian GA (2003). Templates of the cartilage layers of the patellofemoral joint and their use in the assessment of osteoarthritic cartilage damage. Osteoarthr Cartil.

[CR5] Conlan T, Garth WP, Lemons JE (1993). Evaluation of the medial soft-tissue restraint of the extensor mechanism of the knee. J Bone Joint Surg Am.

[CR6] Desio SM, Burks RT, Bachus KN (1998). Soft tissue restraints to lateral patellar translation in the human knee. Am J Sports Med.

[CR7] DeVries WNA, Duchman KR, Bollier MJ (2015). A finite element analysis of medial patellofemoral ligament reconstruction. Iowa Orthop J.

[CR8] Drez D, Edwards TB, Williams CS (2001). Results of medial patellofemoral ligament reconstruction in the treatment of patellar dislocation. Arthroscopy.

[CR9] Elias JJ, Bratton DR, Weinstein DM, Cosgarea AJ (2006). Comparing two estimations of the quadriceps force distribution for use during patellofemoral simulation. J Biomech.

[CR10] Elias JJ, Cech JA, Weinstein DM, Cosgarea AJ (2005). Reducing the lateral force acting on the patella does not consistently decrease patellofemoral pressures. Am J Sports Med.

[CR11] Elias JJ, Cosgarea AJ (2006). Technical errors during medial patellofemoral ligament reconstruction could overload medial patellofemoral cartilage: a computational analysis. Am J Sports Med.

[CR12] Elias JJ, Kelly MJ, Smith KE, Gall KA, Farr J (2016). Dynamic simulation of the effects of graft fixation errors during medial patellofemoral ligament reconstruction. Orthop J Sports Med.

[CR13] Farahmand F, Senevongse W, Amis A (1998). Quantitative study of the quadriceps muscles and trochlear groove geometry related to instability of the patellofemoral joint. J Orthop Res.

[CR14] Fernandez J, Hunter P, Shim V, Mithraratne K, Calvo Lopez B, Peña E (2012). A subject-specific framework to inform musculoskeletal modeling: outcomes from the IUPS physiome project. Patient-specific computational modeling. Lecture notes in computational vision and biomechanics.

[CR15] Fink C, Veselko M, Herbort M, Hoser C (2014). MPFL reconstruction using a quadriceps tendon graft: part 2: operative technique and short term clinical results. Knee.

[CR16] Fulkerson JP, Edgar C (2013). Medial quadriceps tendon-femoral ligament: surgical anatomy and reconstruction technique to prevent patella instability. Arthrosc Tech.

[CR17] Good L (1995). In vitro correlation between tension and length change in an anterior cruciate ligament substitute. Clin Biomech (Bristol, Avon).

[CR18] Hautamaa PV, Fithian DC, Kaufman KR, Daniel DM, Pohlmeyer AM (1998). Medial soft tissue restraints in lateral patellar instability and repair. Clin Orthop Relat Res.

[CR19] Jones B, Hung CT, Ateshian G (2016). Biphasic analysis of cartilage stresses in the patellofemoral joint. J Knee Surg.

[CR20] Martí-Bonmatí L, Sanz-Requena R, Alberich-Bayarri A (2008). Pharmacokinetic MR analysis of the cartilage is influenced by field strength. Eur J Radiol.

[CR21] Moritomo H, Noda K, Goto A, Murase T, Yoshikawa H, Sugamoto K (2009). Interosseous membrane of the forearm: length change of ligaments during forearm rotation. J Hand Surg Am.

[CR22] Rood A, Hannink G, Lenting A, Groenen K, Koëter S, Verdonschot N, van Kampen A (2015). Patellofemoral pressure changes after static and dynamic medial patellofemoral ligament reconstructions. Am J Sports Med.

[CR23] Sanchis-Alfonso V (2014). Guidelines for medial patellofemoral ligament reconstruction in chronic lateral patellar instability. J Am Acad Orthop Surg.

[CR24] Sanchis-Alfonso V (2016). How to deal with chronic patellar instability: what does the literature tell us?. Sports Health.

[CR25] Sanchis-Alfonso V, Ramirez-Fuentes C, Montesinos-Berry E, Domenech J, Martí-Bonmati L (2017). Femoral insertion site of the graft used to replace the medial patellofemoral ligament influences the ligament dynamic changes during knee flexion and the clinical outcome. Knee Surg Sports Traumatol Arthrosc.

[CR26] Segal NA, Anderson DD, Iyer KS (2009). Baseline articular contact stress levels predict incident symptomatic knee osteoarthritis development in the MOST cohort. J Orthop Res.

[CR27] Seo YJ, Song SY, Kim IS, Seo MJ, Kim YS, Yoo YS (2014). Graft tension of the posterior cruciate ligament using a finite element model. Knee Surg Sports Traumatol Arthrosc.

[CR28] Servien E, Fritsch B, Lustig S (2011). In vivo positioning analysis of medial patellofemoral ligament reconstruction. Am J Sports Med.

[CR29] Shah KS, Saranathan A, Koya B, Elias JJ (2015). Finite element analysis to characterize how varying patellar loading influences pressure applied to cartilage: model evaluation. Comput Methods Biomech Biomed Engin.

[CR30] Smirk C, Morris H (2003). The anatomy and reconstruction of the medial patellofemoral ligament. Knee.

[CR31] Stephen JM, Kaider D, Lumpaopong P, Deehan DJ, Amis AA (2014). The effect of femoral tunnel position and graft tension on patellar contact mechanics and kinematics after medial patellofemoral ligament reconstruction. Am J Sports Med.

[CR32] Stephen JM, Kittl C, Williams A, Zaffagnini S, Marcheggiani GM, Fink C, Amis AA (2016). Effect of medial patellofemoral ligament reconstruction method on patellofemoral contact pressures and kinematics. Am J Sports Med.

[CR33] Tan J, Xu J, Xie RG, Deng AD, Tang JB (2011). In vivo length and changes of ligaments stabilizing the thumb carpometacarpal joint. J Hand Surg Am.

[CR34] Teitge RA, Torga-Spak R (2004). Medial patellofemoral ligament reconstruction. Orthopedics.

[CR35] Weinberger JM, Fabricant PD, Taylor SA, Mei JY, Jones KJ (2017). Influence of graft source and configuration on revision rate and patient-reported outcomes after MPFL reconstruction: a systematic review and meta-analysis. Knee Surg Sports Traumatol Arthrosc.

