# Supplementary material for: Parametric finite element model of medial patellofemoral ligament reconstruction model development and clinical validation
Source: J Exp Orthop. 2019 Jul 5;6:32. doi: 10.1186/s40634-019-0200-x (PMC6611858; doi:10.1186/s40634-019-0200-x)
Supplement: Supplementary file 1 — Figure S1. Patellar cartilage contact pressure (MPa) obtained for different values of friction coefficient (µ) (0.01, 0.02, 0.025, 0.03) in the 30º knee flexion angle for the native knee model. Figure S2. Patellar cartilage contact pressure (MPa) obtained for different patellar-femoral cartilages combinations in the 30º knee flexion angle for the intact knee model. PCT=Patellar Cartilage Thickness, FCT=Femoral Cartilage Thickness. (DOCX 387 kb) [file 40634_2019_200_MOESM1_ESM.docx]

Supplementary material for the manuscript entitled *"Parametric Finite Element Model of Medial Patellofemoral Ligament Reconstruction. Model Development and Clinical Validation"*

**Sensitivity analysis of the friction coefficient (µ)**

The value selected for the friction coefficient (µ) for the parametric finite element analysis developed in this manuscript was set to 0.02 (Besier et al. 2008). The influence of this parameter on the contact pressure when its value was changed to 0.01, 0.025 and 0.03 was represented in Figure 1. The sensitivity analysis was performed for the case of the native knee in a 30º knee flexion angle. Almost no differences were estimated.


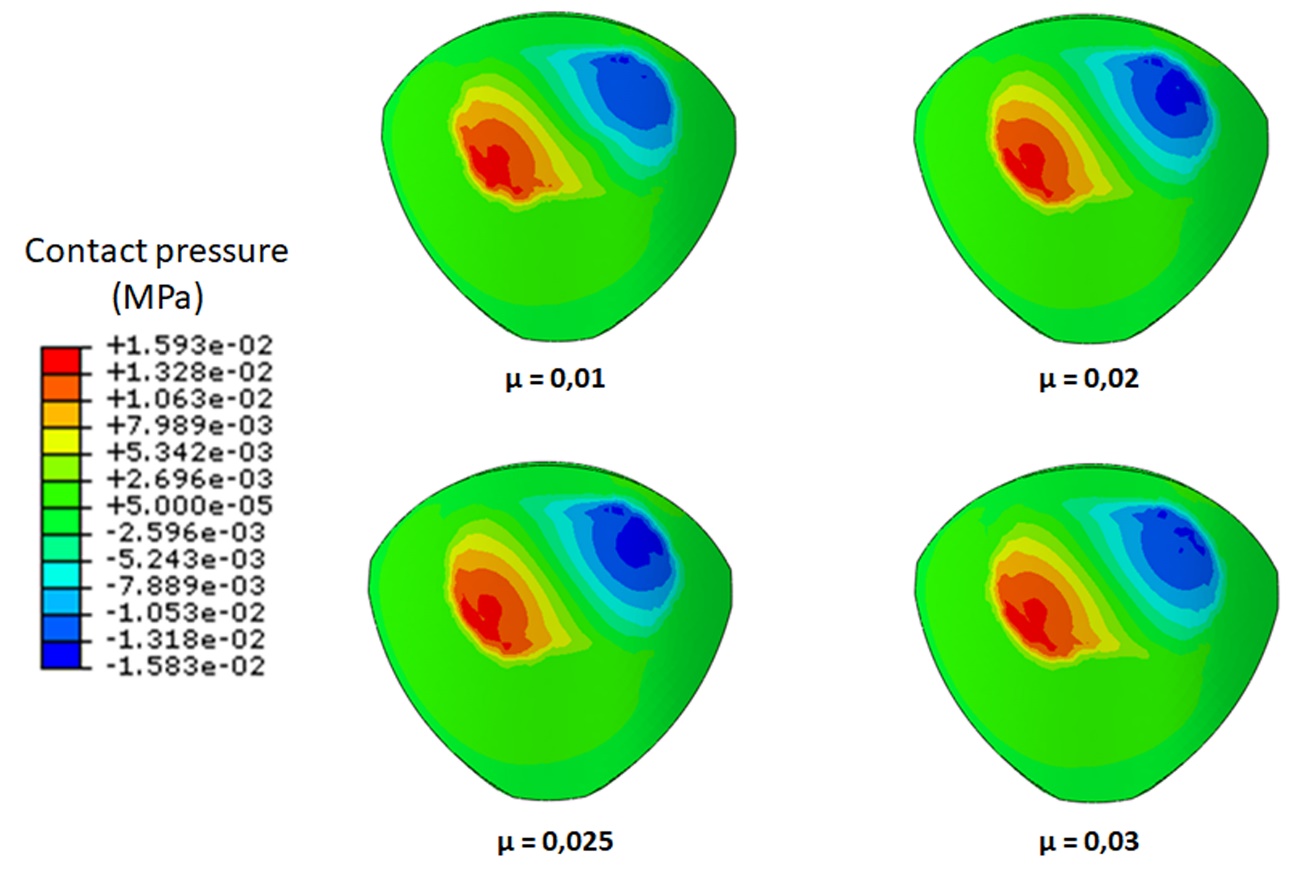


Figure 1. Patellar cartilage contact pressure (MPa) obtained for different values of friction coefficient (µ) (0.01, 0.02, 0.025, 0.03) in the 30º knee flexion angle for the native knee model.

**Sensitivity analysis of the cartilage thickness**

A fixed thickness of 3 mm was assumed for both the patellar and femoral cartilages (Cohen et al., 2003). An analysis changing their corresponding thickness was performed (Figure 2). The differences estimated for the contact pressure distribution were small enough (variation < 0.005 MPa) to assume that cartilage thickness does not influence the overall results.


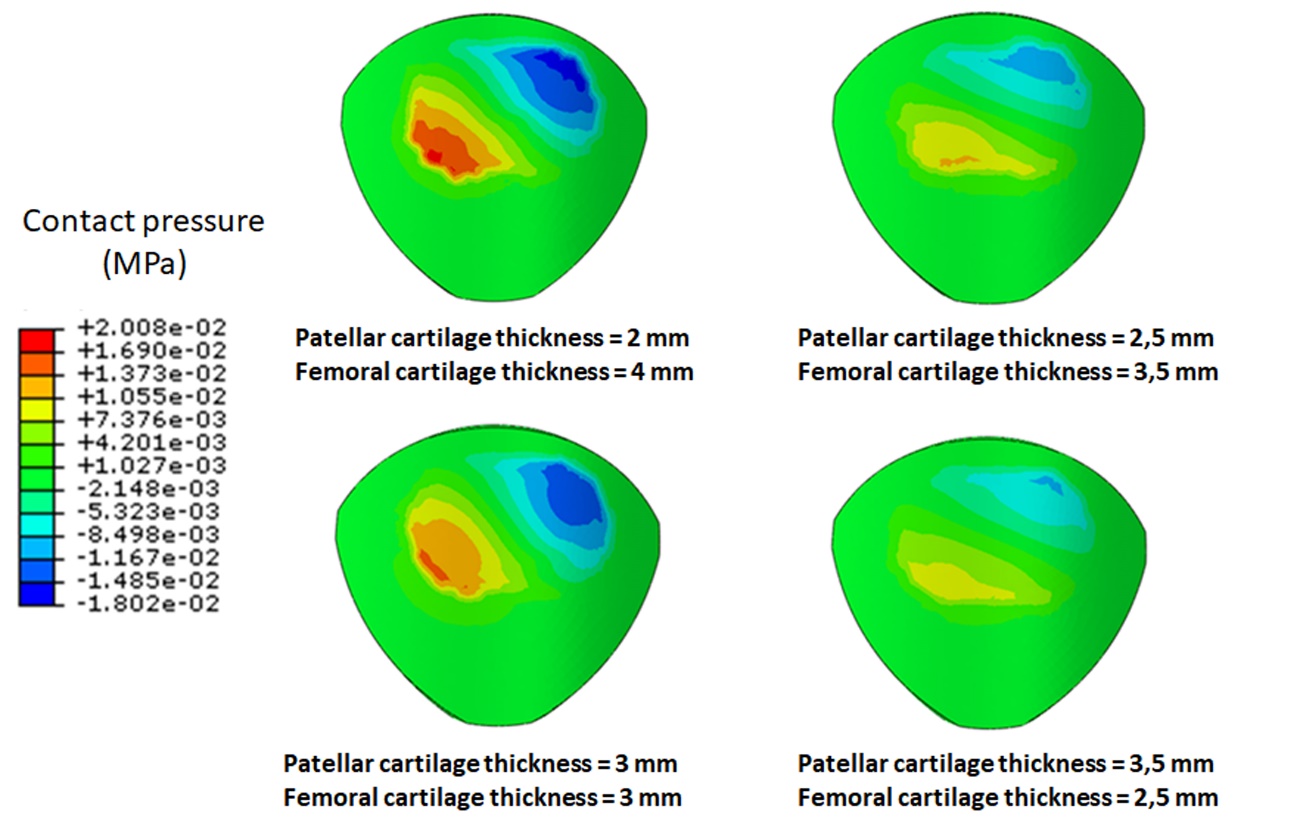


Figure 2. Patellar cartilage contact pressure (MPa) obtained for different patellar-femoral cartilages combinations in the 30º knee flexion angle for the intact knee model. PCT=Patellar Cartilage Thickness, FCT=Femoral Cartilage Thickness

**References**

Besier TF, Gold GE, Delp SL, Fredericson M, Beaupre GS (2008) The influence of femoral internal and external rotation on cartilage stresses within the patellofemoral joint. J Orthop Res 26: 1627-1635

Cohen ZA, Mow VC, Henry JH, Levine WN, Ateshian GA (2003) Templates of the cartilage layers of the patellofemoral joint and their use in the assessment of osteoarthritic cartilage damage. Osteoarthritis Cartilage 11: 569-579
